# Computational Micro-Macro Analysis of Impact on Strain-Hardening Cementitious Composites (SHCC) Including Microscopic Inertia

**DOI:** 10.3390/ma13214934

**Published:** 2020-11-03

**Authors:** Erik Tamsen, Iurie Curosu, Viktor Mechtcherine, Daniel Balzani

**Affiliations:** 1 Institute of Mechanics and Shell Structures, TU Dresden, 01062 Dresden, Germany; 2 Chair of Continuum Mechanics, Ruhr University Bochum, 44801 Bochum, Germany; daniel.balzani@rub.de; 3 Institute of Construction Materials, TU Dresden, 01062 Dresden, Germany; iurie.curosu@tu-dresden.de (I.C.); mechtcherine@tu-dresden.de (V.M.)

**Keywords:** computational homogenization, microscopic inertia, SHCC, ECC, HPFRCC, fiber pullout, impact

## Abstract

This paper presents a numerical two-scale framework for the simulation of fiber reinforced concrete under impact loading. The numerical homogenization framework considers the full balance of linear momentum at the microscale. This allows for the study of microscopic inertia effects affecting the macroscale. After describing the ideas of the dynamic framework and the material models applied at the microscale, the experimental behavior of the fiber and the fiber–matrix bond under varying loading rates are discussed. To capture the most important features, a simplified matrix cracking and a strain rate sensitive fiber pullout model are utilized at the microscale. A split Hopkinson tension bar test is used as an example to present the capabilities of the framework to analyze different sources of dynamic behavior measured at the macroscale. The induced loading wave is studied and the influence of structural inertia on the measured signals within the simulation are verified. Further parameter studies allow the analysis of the macroscopic response resulting from the rate dependent fiber pullout as well as the direct study of the microscale inertia. Even though the material models and the microscale discretization used within this study are simplified, the value of the numerical two-scale framework to study material behavior under impact loading is demonstrated.

## 1. Introduction

Strain-hardening cementitious composites (SHCC), also called engineered cementitious composites (ECC), represent a novel type of high-performance fiber reinforced cementitious composites (HPFRCC) consisting of fine grained cementitious matrices and high-performance polymer microfibers typically in a volume content of 2%. The micromechanically regulated material design of SHCC accounts for the mechanical and physical properties of the cementitious matrix, of the reinforcing fibers and of their bond (see [1,2]). This ensures a strain-hardening tensile behavior of SHCC accompanied by the formation of multiple, fine cracks under increasing deformation. The high strain capacity prior to failure localization, notable damage tolerance and outstanding energy dissipation capacity make SHCC promising as the main material for new structural elements and as strengthening layers applied on existing structures subject to earthquake, impact or blast [3,4].

Given the pronounced rate sensitivity of SHCC in terms of tensile strength and strain capacity, a targeted material design for applications involving dynamic loading requires a proper understanding of the governing mechanisms and phenomena at the micro- and mesoscales [5]. Furthermore, the sound upscaling and application of the meso-level findings in the structural design involving SHCC should be supported by adequate numerical scale-linking models. This is due to the fact that the assessment and discrimination of various structural effects as inertia phenomena and complex fracture-mechanical mechanisms is hardly possible based on structural tests alone (see [6,7,8]). Multiscale methods represent a powerful tool to link the mesoscale and structural scale within the corresponding composite material.

The general idea of computational homogenization methods is to use a fine discretization of the microscale as material input for a coarse macroscopic problem. This can reduce the computational cost significantly, while including fine micromechanical details. There are various frameworks that consider the dynamic effects of the microscale. There is the method of asymptotic expansion (e.g., [9,10,11,12]) which is mainly based on the original work of Bensoussan et al. [13] and then the more general theory of elastodynamic homogenization by Willis [14], applied by, e.g., the authors of [15,16,17]. Both methods are limited to elastic, periodic media. A more general approach is the micro-macro simulation based on a representative volume element (RVE). When the finite element method (FEM) is used on both scales in a scale-coupled manner, it is called the FE${}^{2}$ method. A comprehensive introduction to this theory including dynamics was given by de Souza Neto et al. [18]. Within the FE${}^{2}$ method, there are still different approaches, usually optimized to special conditions. The framework in [9] considers a quasi-static microstructure but then applies an additional body force at the macroscale to account for microinertia effects. This framework was extended by Karamnejad and Sluys [19] to account for localizations at the microscale under impact loading. Further FE${}^{2}$ type schemes calculate the full balance of linear momentum at the microscale. In [20], an explicit, periodic, small strain framework is presented for modeling resonant elastic metamaterials. This was extended to an implicit time integration method by Liu and Reina [21]. By splitting the problem into a purely static and a special dynamic boundary value problem (BVP) in [22,23], the assumption of linear elasticity is used to improve the computational performance. To better capture a wider range of applied frequencies, the work of Sridhar et al. [24] uses a Floquet–Bloch transformation to build a base of eigenmodes to analyze elastic, periodic metamaterials.

The framework applied in the article at hand is presented in [25]. It is also of FE${}^{2}$ type, but has a more general approach. The formulations are compatible with standard FE architecture. To enable the analysis of micromechanical processes such as plasticity or fiber pullout, as well as to incorporate effects of geometric nonlinearities, the framework uses a finite-strain formulation. In addition, a kinematic scale link is proposed which would allow the study of arbitrary crack paths. The framework is applicable to lower frequencies, which makes it suitable for impact investigations.

This paper applies the developed homogenization method in [25] to study full sized SHCC specimens under tensile impact loading, while simultaneously including the most relevant microstructural mechanisms, such as matrix cracking and fiber pullout. To the best of the authors knowledge, this is the first application of a multiscale framework which considers microinertia for a simulation of fiber reinforced concrete to study the dynamic effects arising at the microscale. In this paper, the experimental behavior of the constituents under different loading rates are presented first. Subsequently, an overview of the dynamic FE${}^{2}$ framework is given, followed by a detailed discussion of the material models used at the microscale. These aspects are combined into simulations of split Hopkinson tension bar tests on SHCC samples, to showcase the possibilities of a dynamic multiscale analysis.

## 2. Materials and Experimental Results

The microstructure simulated in this paper corresponds to a high-strength SHCC described in detail in previous studies by Curosu and Mechtcherine [26,27]. The cementitious matrix has a fine-grained nature with aggregates consisting of a relatively small content of fine sand with particle sizes between 0.06 and 0.2 mm. The density of the SHCC is approximately 2135 kg/m${}^{3}$ [26,28]. The high-strength SHCC under investigation and the constitutive matrix have a Young’s modulus of 29 GPa, while the compressive strength ranges between 130 and 140 MPa [26,27,28]. The tensile strength of the cementitious matrix under quasi-static loading is approximately 3.4 MPa [27]. The use of high-performance polymer micro-fibers in SHCC, such as ultra-high-molecular-weight polyethylene (UHMWPE, shortened to PE), is motivated by their small diameter, high tensile strength, moderate Young’s modulus and relatively high elongation capacity. All these properties are beneficial for strain-hardening and steady-state cracking [2]. The PE fibers in the high-strength SHCC under consideration have an average diameter of 20 μm and a cut-length of 12 mm. The nominal tensile strength as provided by the producer is approximately 2500 MPa, the Young’s modulus is 80 GPa and the elongation at break is 3.5%. The PE fibers exhibit a hydrophobic nature and no chemical adhesion to cementitious matrices [28]. Their bond is of frictional nature, whereas their uneven surface texture ensures additionally a mechanical interlock during crack-bridging [5]. The geometric, surface and mechanical properties of the applied PE fibers enable a controlled pullout behavior, and a balanced post-peak softening behavior of SHCC under tension [28].

**Figure 1:**
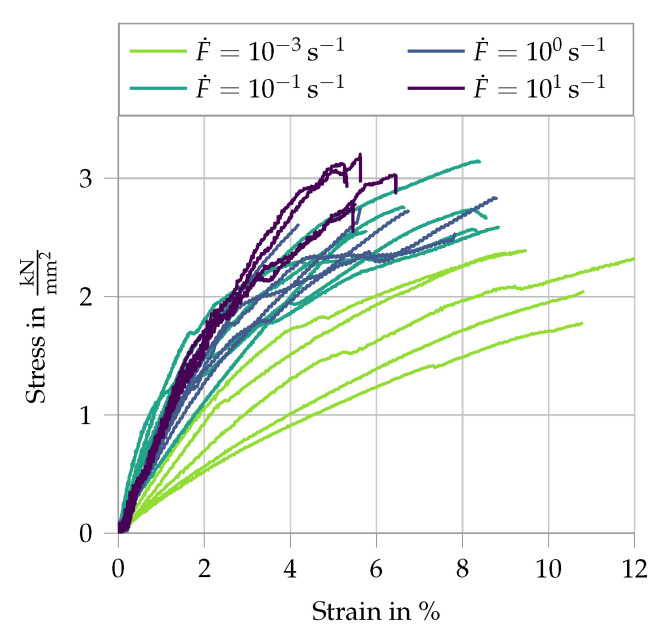
Results of PE fiber tension tests at different strain rates; data from [5,26].

**Figure 2:**
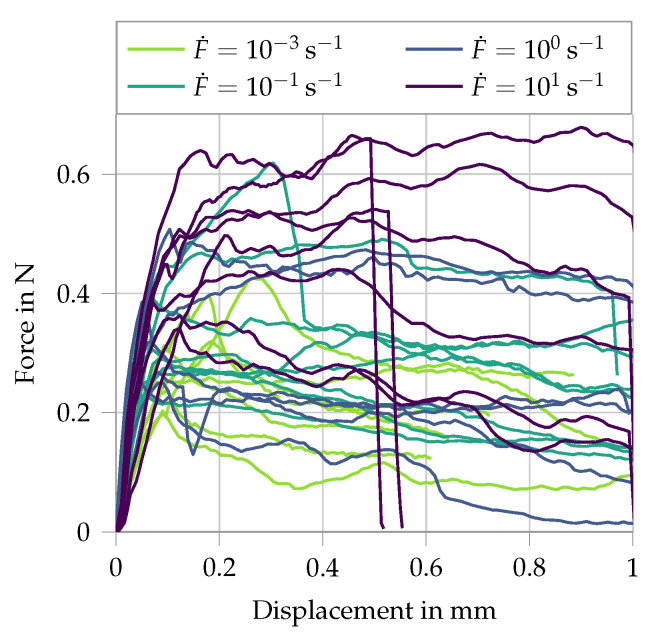
Results of PE fiber in pullout tests at different displacement/loading rates; data from [5,26].

The mechanical properties of the fibers were assessed in single-fiber pullout experiments, performed in an amplified piezoelectric actuator at displacement rates ranging from 0.005 to 50 mm/s (strain rates between 0.001 and 1 s${}^{-1}$). The free length of the fibers in the tension experiments was 5 mm [5,26]. Additionally, pullout tests were performed in the same testing setup under identical displacement rates as the single-fiber tension tests, whereas the embedment length was 2 mm. For details regarding specimen preparation and testing configuration, see [5]. The single-fiber tension experiments demonstrated a pronounced non-linear tensile behavior and rate-dependent tensile strength, Young’s modulus and elongation capacity of the PE fibers. At higher displacement rates, the fibers yielded an increase in tensile strength and Young’s modulus, but a decrease in elongation capacity (see Figure 1). The results of the single-fiber pullout experiments are presented in Figure 2. Note that the stroke in the testing device was limited to 1 mm and the fibers could not be pulled out completely from the matrix specimens. The entire pullout pattern for this fiber–matrix combination under quasi-static loading can be found in [28]. The pullout curves show an increase in bond strength and slip-hardening degree with increasing displacement rate. Given the frictional bond and mechanical anchorage of the hydrophobic fibers in the cementitious matrix, the increased pullout resistance with increasing displacement rates was traced back to the rate-sensitive Young’s modulus of the fibers. Assuming a rate independent Poisson’s ratio, the dynamically enhanced Young’s modulus limited the radial contraction of the loaded fibers and consequently the reduction in interfacial confinement during pullout.

Both the single-fiber tension and pullout experiments served as experimental basis for the calibration of the micromechanical parameters in the developed numerical model. Due to the limitations imposed by the testing facilities and measuring techniques, the micromechanical experiments implied displacement rates considerably lower than the crack opening speeds in SHCC subject to tensile impact loading in Hopkinson bar tests [5,29], which served for validation purposes in the presented numerical study. Thus, for the modeling of SHCC, the derived micromechanical parameters at higher strain/displacement rates were extrapolated assuming a linear rate dependency. The discrepancy between these assumptions and the effective rate dependencies could be assessed in the numerical parameter study presented in Section 5.

## 3. Numerical Two-Scale Framework Accounting for Microscopic Inertia

This section provides an overview of the assumptions the applied numerical multiscale framework is based on and presents the resulting formulations necessary for an implementation of the homogenization scheme. A more detailed description of the full derivation is given in [25]. The proposed framework is an FE${}^{2}$ homogenization method with the key characteristic that it considers the full balance of linear momentum at the microscale. This enables the direct analysis of full dynamic fields at the microscale, while simultaneously allowing the study of resulting effects at the macroscale. Depicted is the two-way coupling, where each macroscopic integration point is associated with a separate microscopic RVE simulation. The macroscopic values are required as input for the microscopic boundary value problem (BVP). By using appropriate averaging relations and kinematic links, a consistent scale bridging for dynamic loading is established. After solving for microscopic equilibrium, the homogenized fields of stress $\bar{P}$ (here in terms of the first Piola–Kirchhoff stress tensor) and the homogenized inertia force vector ${\bar{f}}^{\rho }$ are passed to the macroscopic problem, along with four essential tangent moduli ${\bar{A}}^{P\;,F}$, ${\bar{A}}^{P\;,u}$, ${\bar{A}}^{f\;,F}$ and ${\bar{A}}^{f\;,u}$, representing the derivatives of stress and body force with respect to deformation gradient $\bar{F}$ and displacement $\bar{u}$, respectively. In the following, values associated with the macroscale are indicated by a bar $\bar{•}$. To enable the analysis of versatile micromechanical phenomena, a finite-strain formulation is used. The (undeformed) reference and the (deformed) current configuration are linked by the displacement u=x−X, where X∈B refers to the coordinates in the undeformed reference configuration and x∈S to the coordinates in the deformed configuration. The transformation between the configurations in terms of vector elements is described by the deformation and displacement gradients, respectively, $F=\partial_{X}x=1+H$ and $H=\partial_{X}u$, such that x=FX. To simplify the notation, the origin of the microscopic coordinates is chosen as the geometrical center of the RVE, with $\int_{B}X\;dV=0$. This choice has no influence on the results. Under the assumption of scale separation, the microscopic deformation x can be split into a sum of terms,

(1) $$\begin{array}{c} x=\bar{u}+\bar{F}X+\tilde{u}. \end{array}$$

Two terms result directly from the macroscale: a constant part $\bar{u}$, which describes the macroscopic rigid body translations, and a homogeneous part $\bar{F}X$, defined in terms of the macroscopic deformation gradient. $\tilde{u}$ denotes the microscopic displacement fluctuation field, which is the field the microscopic BVP is solved for. Analogously, the microscopic deformation gradient can be written as

(2) $$\begin{array}{c} F=\bar{F}+\tilde{H}\;\mathrm{with}\;\tilde{H}=\partial_{X}\tilde{u}. \end{array}$$

To account for the microscale dynamics, an extended version of the Hill–Mandel condition of macro homogeneity also called the Principle of Multiscale Virtual Power is adopted, as base for the derivation of the framework (cf. [18,30]). It ensures that the virtual work of the macroscale coincides with its respective microscopic volume average. The resulting averaging equation for the effective macroscopic stress $\bar{P}$ and the effective macroscopic body force vector $\bar{f}$ are given as

(3) $$\begin{array}{c} \bar{P}=\langle P-f⊗X\rangle \;\mathrm{and}\;\bar{f}=\langle f\rangle . \end{array}$$

Herein, $\langle •\rangle =\frac{1}{V}\int_{B}•\;dV$ is an abbreviation for the volume average of a microscopic quantity. A principal ingredient of the Hill–Mandel condition is the assumption of a clear separation of scales. This stipulates that the fluctuations of mechanical fields at the microscale must be significantly smaller than those of the macroscopic problem. For dynamic homogenization, this signifies in practice that additionally the principal wavelength of the applied macroscopic loading must be sufficiently larger than the size of the RVE. For dealing with time derivatives, the Newmark method [31] is applied—a widely used implicit numerical time integration method of first order. First, the microscopic element formulations are viewed in more detail. Subsequently, the respective macroscopic element equations are given.

### 3.1. The Microscopic Problem

To allow a full dynamic analysis at the microscale, the microscopic balance of linear momentum is given by

(4) $$\begin{array}{c} \mathrm{Div}P+f=0. \end{array}$$

This paper models impact loading, where gravitational forces are negligible compared to the inertia forces. Therefore, only the inertia part of the body forces $f^{\rho }$ is considered here. The relevant body force vector is defined as $f:=f^{\rho }=-\rho_{0}\ddot{u}$ with $\rho_{0}$ denoting the density of the microscale components in the undeformed configuration. Following the standard FEM algorithm, the global tangent stiffness matrix $\hat{K}$ is assembled from the element matrix,

(5) $$\begin{array}{cc} \; & {\hat{k}}^{e}=k^{e}+\frac{1}{\beta \Delta t^{2}}m^{e},\;\mathrm{with} \end{array}$$

(6) $$\begin{array}{cc} k^{e}=\int_{B^{e}}B^{e^{T}}\; & AB^{e}\;dV\;\mathrm{and}\;m^{e}=\int_{B^{e}}N^{e}\;\rho_{0}N^{e^{T}}\;dV, \end{array}$$

where $N^{e}$ is the classical element matrix of shape functions, $B^{e}$ denotes the B-matrix containing the derivatives of the shape functions and A is the matrix representation of the material tangent modulus, defined as $A=\partial_{F}P$. β is one of the two Newmark parameters. Throughout this work, the parameters are set as β=0.25 and γ=0.5. Analogously to the stiffness matrix, the global residuum matrix $\hat{R}$ is obtained by the assembly of the element-wise counterparts in matrix representation,

(7) $$\begin{array}{c} {\hat{r}}^{e}=\int_{B^{e}}\left(B^{e^{T}}\;P+N^{e}\;\rho_{0}\ddot{u}\right)\;dV. \end{array}$$

After including Dirichlet boundary conditions, the resulting discrete system of equations at the microscale reads

(8) $$\begin{array}{c} \hat{K}\Delta \tilde{D}=\hat{R}. \end{array}$$

The macroscopic displacements and deformation gradient and their time derivatives are used to define boundary conditions on the RVE (cf. (1)). To ensure a consistent application of the macroscopic values, certain constraints need to be enforced at the microscale. The first kinematic link concerns the deformation gradient, $\bar{F}=\langle F\rangle$. It postulates that the volume average of the microscopic deformation gradient must be equal the deformation gradient at the macroscale. This is prescribed by applying periodic boundary conditions on the RVE. To enable a dynamic framework, it needs to be ensured that no arbitrary rigid body motions are possible. Within this work, a simple solution is used for the second kinematic constraint. By applying the macroscopic displacements $\bar{u}$ to the corners of the RVE and simultaneously forcing the microscopic displacement fluctuations to be zero, a direct coupling of the macroscopic displacements is achieved. This constraint is chosen a priori and might not be optimal for all problems; however, for the problem at hand, it is well suited. A softer and more general constraint which applies the macroscopic displacements as the volume average using Lagrange multipliers is presented in [25].

### 3.2. The Macroscopic Problem

After identifying the formulations at the microscale based on macroscopic values, the bilateral coupling is realized by defining the respective macroscopic values depending on the microscopic fields (cf. (3)). In the same way as for the microscale, the complete macroscopic balance of linear momentum including inertia is considered. Here, the weak form of linear momentum is given without the contributions of external traction as,

(9) $$\begin{array}{c} \bar{G}:=\int_{\bar{B}}\delta \bar{F}:\bar{P}\;dV+\int_{\bar{B}}\delta {\bar{u}}^{T}{\bar{f}}^{\rho }\;dV=0. \end{array}$$

Once more, only body forces related to inertia are regarded, such that $\bar{f}:={\bar{f}}^{\rho }=\langle f^{\rho }\rangle$. To apply the standard Newton–Raphson scheme, the linearized balance of linear momentum is obtained as

(10) $$\begin{array}{c} \mathrm{Lin}\bar{G}=\bar{G}+\Delta \bar{G}=0\;\mathrm{with}\;\Delta \bar{G}=\int_{\bar{B}}\delta \bar{F}:\Delta \bar{P}\;dV+\int_{\bar{B}}\delta {\bar{u}}^{T}\Delta {\bar{f}}^{\rho }\;dV. \end{array}$$

Within the multiscale framework, the macroscopic values of stress $\bar{P}$ and inertia ${\bar{f}}^{\rho }$ are defined in terms of the microscale. Furthermore, the microscopic fields depend on both the macroscopic acceleration $\ddot{\bar{u}}$ as well as the macroscopic deformation gradient $\bar{F}$. Therefore, the effective macroscopic stress and inertia for any given point at the macroscale are sensitive to both its displacement as well as the respective deformation gradient. This is a special property arising from the consideration of the microscale dynamics. From this observation, it follows directly that the linearized terms are expanded as

(11) $$\begin{array}{c} \Delta \bar{P}=\frac{\partial \bar{P}}{\partial \bar{F}}:\Delta \bar{F}+\frac{\partial \bar{P}}{\partial \ddot{\bar{u}}}\cdot \Delta \ddot{\bar{u}}\;\mathrm{and}\;\Delta {\bar{f}}^{\rho }=\frac{\partial {\bar{f}}^{\rho }}{\partial \bar{F}}:\Delta \bar{F}+\frac{\partial {\bar{f}}^{\rho }}{\partial \ddot{\bar{u}}}\cdot \Delta \ddot{\bar{u}}. \end{array}$$

The four emerging sensitivities are defined as

(12) $$\begin{array}{c} {\bar{A}}^{P\;,F}\;=\partial_{\bar{F}}\bar{P},\;{\bar{A}}^{P\;,u}\;=\partial_{\ddot{\bar{u}}}\bar{P},\;{\bar{A}}^{f\;,F}\;=\partial_{\bar{F}}{\bar{f}}^{\rho }\;\mathrm{and}\;{\bar{A}}^{f\;,u}\;=\partial_{\ddot{\bar{u}}}{\bar{f}}^{\rho }. \end{array}$$

By applying standard FE discretization to the linearized weak form (10) while considering (11), the macroscopic element stiffness matrix ${\hat{\bar{k}}}^{e}\;$ and the element residuum vector ${\hat{\bar{r}}}^{e}\;$ are identified. Here, the matrix representation of the moduli in index notation is used, where lowercase indices refer to the spacial dimension $n_{\mathrm{dm}}$ and uppercase indices to the total degrees of freedom of an element $n_{\mathrm{edf}}$. This yields the definition of the full macroscopic element matrices as

(13) $$\begin{array}{c} {\hat{\bar{k}}}_{PQ}^{e}=\int_{B^{e}}\left({\bar{B}}_{ijP}^{e}{\bar{A}}_{ijmn}^{P\;,F}{\bar{B}}_{mnQ}^{e}+\frac{1}{\bar{\beta }\Delta t^{2}}{\bar{B}}_{ijP}^{e}{\bar{A}}_{ijk}^{P\;,u}{\bar{N}}_{Qk}^{e}\right.\\ \left.\;\;\;\;\;\;\;+{\bar{N}}_{Pi}^{e}{\bar{A}}_{imn}^{f\;,F}{\bar{B}}_{mnQ}^{e}+\frac{1}{\bar{\beta }\Delta t^{2}}{\bar{N}}_{Pi}^{e}{\bar{A}}_{ik}^{f\;,u}{\bar{N}}_{Qk}^{e}\right)\;dV\;\mathrm{and} \end{array}$$

(14) $$\begin{array}{cc} {\hat{\bar{r}}}_{P}^{e}=\int_{B^{e}} & \left({\bar{B}}_{ijP}^{e}{\bar{P}}_{ij}+{\bar{N}}_{Pi}^{e}{\bar{f}}_{i}^{\rho }\right)\;dV. \end{array}$$

By inserting the averaging equations into the definitions of the moduli and evaluating the linearized weak form of the microscale at equilibrium, the four closed form expressions can be identified in terms of the microscopic fields. This allows an efficient numerical algorithm. The macroscopic tangent moduli have been presented in [25,32].

## 4. Micromechanical Material Models

To present the capability of the dynamic multiscale framework of analyzing microstructures under impact loading, micromechanical models must be implemented that display the most important characteristics. For SHCC, this involves the modeling of the matrix, including a possibility to include cracks as well as the implementation of a fiber pullout mechanism.

### 4.1. SHCC Matrix

As the relevant loading considered within this work is tension, the complex behavior of the matrix under compression can currently be neglected. Therefore the elastic Neo-Hooke material law is used for the matrix material. The Neo-Hookean constitutive law yields a simple expression to model elastic material behavior at large strains. The first Piola–Kirchhoff stress tensor is given as

(15) $$P=\;\left(\lambda \;\ln\;\left[\;\det[F]\;\right]-\mu \right)F^{-T}+\mu F,$$

with the Lamé constants λ and μ. For more details, see, e.g., the work of Bonet and Wood [33]. In the tensile regime, the crack development is the most important mechanical mechanism of the matrix. The simulation of proper crack propagation is a highly complex field and not in the scope of the current work. As an approximation, a simple erosion technique is implemented for the matrix. Once the specified matrix material reaches a stress threshold $\sigma_{\mathrm{cr}}$ in loading direction, its stiffness is reduced to a small value $E_{\mathrm{cr}}$, resulting in an effective crack. As the threshold is evaluated at the local material point, this method is mesh dependent. Therefore, only simulations using the same microscopic mesh are directly compared. Due to the approximation of the fibers as truss elements directly connected to the matrix nodes, this erosion method cannot be applied evenly to all matrix elements, as artificial stress localization at the shared nodes would lead to a non-physical erosion of the fiber anchorages. Consequently, the crack location needs to be defined before the computation.

### 4.2. Effective Fiber Pullout

The full fiber pullout is represented by a linear truss element. The effective material model consists of a general 1D Neo-Hookean material law with two additional features. The model includes a strain rate sensitivity, as observed in the micromechanical tests of the fiber material, and a damage formulation to represent the pullout behavior (cf. Section 2). It is based on the simplified assumption that the fibers are engaged by a crack crossing it in the center, leading to the same pullout function for all fibers. The standard 3D Neo-Hookean stress formulation can be simplified to 1D as

(16) $$\begin{array}{c} P=\frac{1}{2}E\left(F-\frac{1}{F}\right), \end{array}$$

where *E* is the Young’s modulus. This is then extended with a multiplicative approach to include a damage and strain rate formulation as

(17) $$\begin{array}{c} \hat{P}=P\left(1+\Omega \right)\left(1-D\right). \end{array}$$

Here, Ω denotes the dynamic increase, which takes on only positive values. It is defined using a logarithmic function of the rate of the deformation gradient, as

(18) $$\Omega =\left\{\begin{array}{cc} \alpha^{I}\;\ln\;\left[\frac{\dot{F}}{\alpha^{\mathrm{II}}}\right]\;\;\; & \dot{F}\geq \alpha^{\mathrm{II}}\\ 0\; & \dot{F}<\alpha^{\mathrm{II}} \end{array}\right..$$

The two parameters $\alpha^{I}$ and $\alpha^{\mathrm{II}}$, respectively, determine the slope and zero value of the logarithmic function. This allows an increase in stress for high deformation rates. The damage formulation is the governing mechanism to represent the effective fiber pullout. The scalar parameter *D* takes on values of 0 to 1, where 1 represents a fully damaged state, in this case a full fiber pullout. Applying the strain equivalence principle, an exponential damage function has been chosen as

(19) $$\begin{array}{c} D=D_{\infty }\left(1-\exp\left(-{\left(\frac{\psi_{D}}{D_{\mathrm{rate}}}\right)}^{D_{\mathrm{shape}}}\right)\right). \end{array}$$

The damage value *D* is determined based on the internal variable $\psi_{D}$, representing the effective energy considered for damage.

It is defined as the maximum value of the strain energy function $\psi_{0}$ which has so far been reached. Thus, the damage evolves only when $\psi_{0}>\psi_{D}$. This results in a discontinuous damage approach. There are three material parameters associated with the damage formulation. $D_{\infty }$ defines the maximum reachable damage value. The model parameter $D_{\mathrm{rate}}>0$ influences the velocity of the damage evolution, $D_{\mathrm{shape}}$ enables the modification of the overall shape of the function, where values below 1 will increase the damage rate at the beginning, while decreasing it for larger deformations.

**Figure 3:**
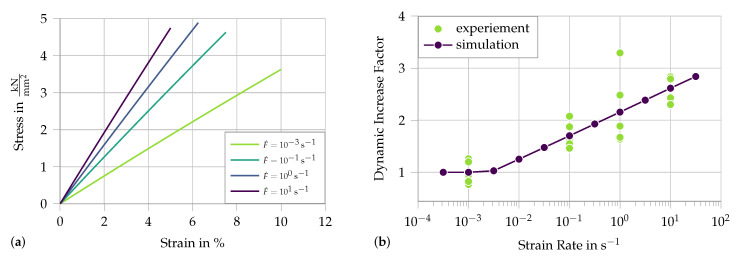
Simulation of single-fiber tension tests with rate-dependent material properties: (**a**) stress–strain diagram; and (**b**) the dynamic increase factor of the secant stiffness at fracture compared to experimental results from [5]. Material parameters used: E=50 kN/mm${}^{2}$, $\alpha^{I}=0.19$ and $\alpha^{\mathrm{II}}=1.8\times 10^{-3}$.

First, we analyze the strain rate effect without the damage formulation, modeling a simple dynamic fiber tension test, as presented in Section 2. Figure 3a presents the stress strain curve of the 1D Neo-Hookean material for different strain rates. To compare the simulation to the experiment, the secant modulus at maximum strain is plotted against the applied strain rate in Figure 3b. The linear increase in stress plotted on a logarithmic scale is clearly visible.

**Figure 4:**
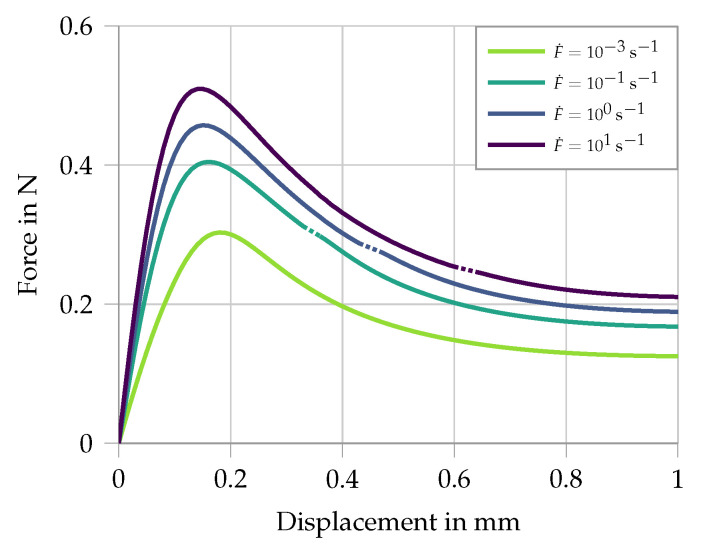
Simulation of single-fiber pullout tests for different strain rates. Material parameters for the free length: E=50 kN/mm${}^{2}$, $\alpha^{I}=0.19$ and $\alpha^{\mathrm{II}}=1.8\times 10^{-3}$. Material parameters for the embedded fiber: E=300 kN/mm${}^{2}$, $\alpha^{I}=0.08$, $\alpha^{\mathrm{II}}=1.8\times 10^{-3}$, $D_{\infty }=0.998$, $D_{\mathrm{rate}}=2.0$ and $D_{\mathrm{shape}}=0.2$.

Secondly, the damage formulation is activated and fitted to the presented single-fiber pullout tests in Figure 2. The simulation uses two elements, one representing the free length of the fiber where only the rate sensitivity is active, and the second element represents the embedded part of the fiber where the pullout takes place. Here both the strain rate sensitivity as well as the damage is active. The stress–strain curves for four different strain rates applied at the boundary are given in Figure 4. Comparing this to the experimental results shows that the overall phases of debonding and pullout are captured. The experiments under higher strain rates show a gradual shift from slip-softening to slip-hardening (see Figure 2). This phenomenon has not been included in the model.

## 5. Numerical Examples

The last sections describe the multiscale framework as well as the material models created for the microscale simulation. This section combines the two by using a well-known experimental setup as example problem: the split Hopkinson tension bar. The present numerical study is not intended as a validation of the framework. This has been conducted previously on other structures (e.g., [25]). First, the experimental setup and results are discussed. Then, a quasi static calculation is used to calibrate the numerical material parameters. Finally, the full split Hopkinson bar simulation is presented and used to show the possibilities of the multiscale framework to perform dynamic simulations.

### 5.1. Split Hopkinson Tension Bar Experiment

**Figure 5:**
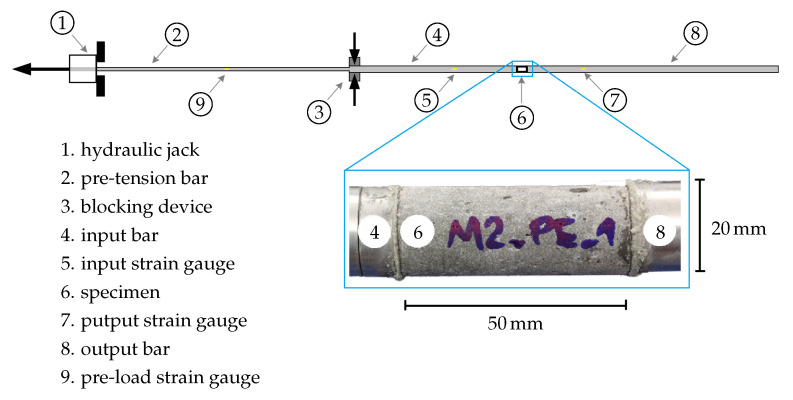
Split Hopkinson tension bar setup, based on [5,27].

**Figure 6:**
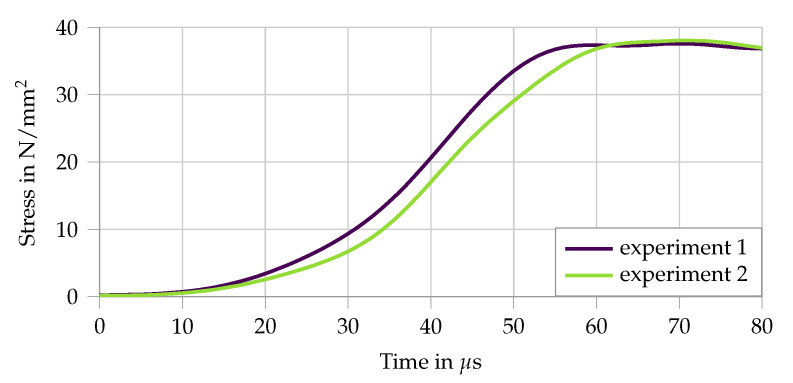
The characteristic trapezoidal loading pulse of the modified split Hopkinson tension bar, as measured in the in input bar, from [5,27]. Here, only the rise is depicted as the first crack occurs before the plateau is reached.

In the split Hopkinson tension bar setup [27], the specimen is sandwiched between two aluminum bars having front and end surface contact, as visualized in Figure 5. The split Hopkinson tension bar setup consists of an input bar of length 3 m and of an output bar 6 m long; both bars are made of aluminum and have a diameter of 20 mm. A high-strength steel bar 6 m long having a diameter of 12 mm is used as a pre-tensioned bar for generating the loading pulse of trapezoidal shape of 2.4 ms duration and with a rise-time of about 60 μs (see Figure 6).

The maximum displacement speed in the test was 6 m/s and the specimen length was 50 mm, which ensured a peak strain rate of 120 s${}^{-1}$. The application of the elastic, uniaxial stress wave propagation theory to the Hopkinson bar system [34] allows calculation of the forces and the displacements acting on the two faces of the specimen in contact with the input and output bars, respectively. From this, the stress at the two interfaces can be inferred, denoted as $\sigma_{1}$ and $\sigma_{2}$ within this work. The specimen is assumed to reach dynamic stress equilibrium if the force-time responses at both ends (derived in the input and output bar) approach each other. This condition imposes a certain number of wave reverberations inside the specimen before damage initiation (cracking) and it is essential for an accurate derivation of the stress–strain relationships. With a specimen length of only 50 mm in the presented experiments, the dynamic stress equilibrium is reached before first crack formation.

**Figure 7:**
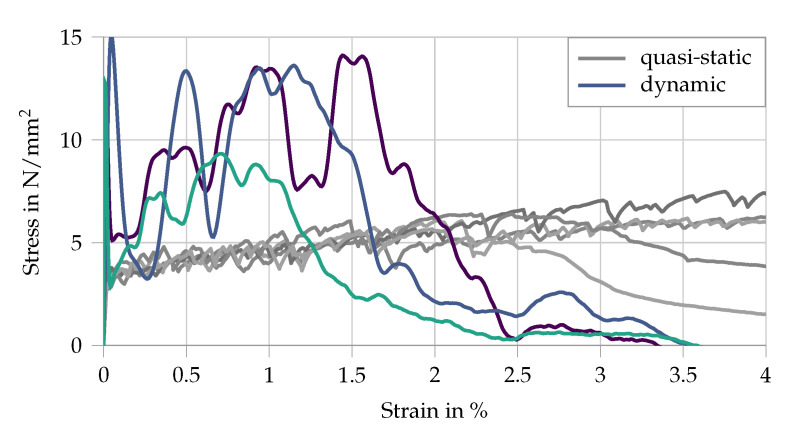
Experimental stress–strain curves for a split Hopkinson bar tension test with SHCC, data from [5,27]. The quasi-static results are given in gray as a comparison.

The resulting stress–strain curves are compared to quasi-static measurements in Figure 7. Damage initiation in the matrix substantially reduces the stiffness of the specimen and the effective strain rate in the matrix. This explains the high initial stress peak and the subsequent multiple cracking occurring at lower stress levels. Moreover, the formation of cracks causes additional wave reflections in the sample, leading to pronounced oscillations of the captured waves in both input and output bars and resulting in an unsteadiness of the derived stress–strain curves, as shown in Figure 7.

Furthermore, the dynamic tensile curves in the split Hopkinson tension bar exhibit higher stresses but lower strains at failure localization, this representing the characteristic rate sensitivity of the high-strength SHCC. The rate-induced reduction of strain capacity can be traced back to a considerably less pronounced multiple cracking compared to quasi-static conditions and is a result of an exaggerated dynamic enhancement of the fiber–matrix bond compared to that of the fiber tensile strength. Such unbalanced micromechanical rate sensitivities lead to a shift from fiber pullout to fiber rupture at higher strain rates, which is disadvantageous with regard to strain-hardening and multiple cracking. This phenomenon cannot be reproduced by the simplified numerical model presented in the paper at hand.

### 5.2. Quasi-Static Simulation

**Figure 8:**
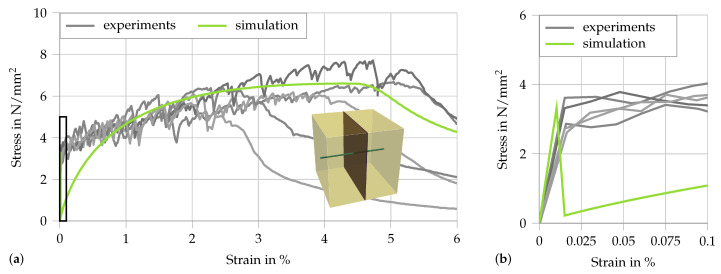
Results of the quasi-static multiscale simulation compared to the experimental data from [5,27]: (**a**) the loading up to 6% strain and the selected RVE; and (**b**) a zoomed-in detail of (**a**), focusing on the cracking of the matrix in the RVEs.

Considering the simplified numerical models discussed in Section 4, there is no gain using a complex microstructure. Processes that would benefit from a detailed microstructure with a realistic distribution and number of fibers, e.g., crack evolution or a fiber pullout behavior depending on the angle to the fracture face, are not within the scope of this paper. Therefore, it is sufficient to discretize the microstructure as a single fiber intersected by a single crack embedded in a cubic RVE with an edge length of 1 mm. The RVE is depicted in Figure 8a.

The mesh consists of three quadratic brick elements, two for the matrix and the one in the center with the possibility for cracking. The embedded fiber is simulated by a single truss element in loading direction. Unfortunately, the micromechanical measurements presented in the last section cannot be directly extrapolated to the case of fully embedded fibers. Therefore, the two-scale SHCC simulation is calibrated by using the results of a quasi-static tension test. To replicate the quasi-static tension experiment, a multiscale simulation is used, which consists of five truss elements at the macroscale. The bar is fixed at one end and a linear displacement load is applied at the other. The stress is recorded at the boundary and the strain is computed as the boundary displacement divided by the specimen length. The following material parameters are applied: for the matrix, E=29 kN/mm${}^{2}$ and ν=0.3; for the crack, $E_{\mathrm{cr}}=10^{-3}$ kN/mm${}^{2}$ and $\sigma_{\mathrm{cr}}=5$ N/mm${}^{2}$; and, for the fiber, E=40 kN/mm${}^{2}$, $A=0.18\;{\mathrm{mm}}^{2}$, $D_{\infty }=0.9982$, $D_{\mathrm{shape}}=0.36$ and $D_{\mathrm{rate}}=0.2$. The resulting stress–strain curve is compared to the experiment in Figure 8, including a zoomed-in section up to the first crack. The overall fit is good; however, instead of a gradual strain-hardening behavior, a sharp drop in stress is observed in the simulation once the first-crack stress $\sigma_{\mathrm{cr}}$ is reached. The theoretical maximum value of $\sigma_{\mathrm{cr}}=5$ N/mm${}^{2}$ is not reached, mainly because of the local nature of the applied erosion method. The phenomenon of the sharp drop in stress is due to the fact that in a quasi-static setting a homogeneous stress state is obtained at the macroscale. Thus, as there is no natural variation in material parameters, all RVEs fracture simultaneously. Nevertheless, after the cracking of the matrix, i.e., when the fibers are engaged, the general debonding behavior matches well that observed in the experiment.

### 5.3. Split Hopkinson Tension Bar Simulation

**Figure 9:**
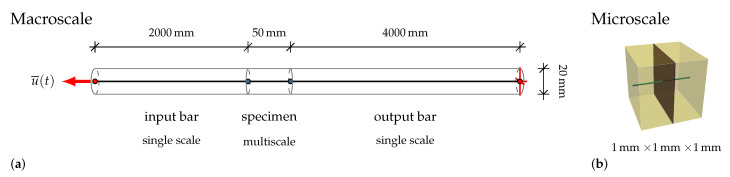
Schematic visualization of the boundary value problem representing the split Hopkinson tension test: (**a**) the macroscopic problem; and (**b**) the discretization of the SHCC microstructure applied in the multiscale simulation of the test specimen.

To properly capture the essential details, first the experimental setup needs to be replicated. A sketch of the macroscopic BVP is given in Figure 9. It consists of a row of truss elements discretized in 10-mm sections.

The input and output bars are simulated with standard single scale elements, whereas the two-scale homogenization framework is used for the SHCC specimen to include the simplified RVEs at the microscale. The next step is the choice of the input load, which is applied via a displacement boundary. Using the recorded signal from the experiments, a piece-wise polynomial function was formulated to represent the loading conditions. It consists of three parts

(20) $$\begin{array}{cc} {\bar{u}}^{I}\left(t\right) & =\frac{14}{275}\;t\;v_{c}{\left(\frac{2t}{t_{\mathrm{vc}}}\right)}^{3}, \end{array}$$

(21) $$\begin{array}{cc} {\bar{u}}^{\mathrm{II}}\left(t\right) & =\frac{t\;v_{c}}{275}\left[7{\left(\frac{2t}{t_{\mathrm{vc}}}\right)}^{8}\;\;-12{\left(\frac{2t}{t_{\mathrm{vc}}}\right)}^{7}\;\;+16{\left(\frac{2t}{t_{\mathrm{vc}}}\right)}^{6}\;\;+19-\frac{34}{3}{\left(\frac{2t}{t_{\mathrm{vc}}}\right)}^{-1}\right]\;\mathrm{and} \end{array}$$

(22) $$\begin{array}{cc} {\bar{u}}^{\mathrm{III}}\left(t\right) & =v_{c}\left(t-\frac{529}{825}t_{\mathrm{vc}}\right). \end{array}$$

The transitions between the respective functions are at ${\bar{u}}^{I}\left(0.592\;t_{\mathrm{vc}}\right)={\bar{u}}^{\mathrm{II}}\left(0.592\;t_{\mathrm{vc}}\right)$ and ${\bar{u}}^{\mathrm{II}}\left(t_{\mathrm{vc}}\right)={\bar{u}}^{\mathrm{III}}\left(t_{\mathrm{vc}}\right)$, so that the loading function is defined as

(23) $${\bar{u}}^{\mathrm{BC}}\left(t\right)=\left\{\begin{array}{ccc} {\bar{u}}^{I}\left(t\right)\; & \;\;\; & 0\leq t\leq 0.592\;t_{\mathrm{vc}}\\ {\bar{u}}^{\mathrm{II}}\left(t\right)\; & \; & 0.592\;t_{\mathrm{vc}}<t\leq t_{\mathrm{vc}}\\ {\bar{u}}^{\mathrm{III}}\left(t\right)\; & \; & t>t_{\mathrm{vc}} \end{array}\right..$$

**Figure 10:**
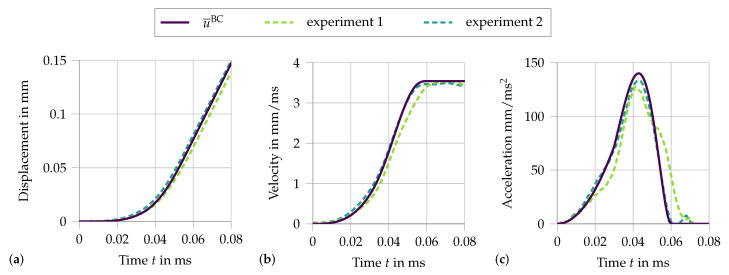
Loading function ${\bar{u}}^{\mathrm{BC}}$ (23) in (**a**), its first time derivative (**b**) and its second time derivative (**c**), compared to the two experiments from [5], with $t_{\mathrm{vc}}=60$ μs and $v_{c}=3540$ mm/s.

Figure 10 compares the chosen displacement function ${\bar{u}}^{\mathrm{BC}}$ and the time derivatives, velocity and acceleration, to two experimental measurements.

Here, the significance of the two loading parameters is visible. The first parameter $t_{\mathrm{vc}}$ defines the time when the transition from the acceleration phase to the phase of constant velocity is completed. The second parameter $v_{c}$ sets the constant velocity. These parameters are easily identified from the experimental data. Even though the experimental data could directly be used as an input at the boundary, the function has the advantage that parameter studies can be easily conducted to analyze the influence of the loading conditions on the specimen’s response. In addition to the adjusted material parameters used in the quasi-static calculation, further parameters accounting for the dynamics must be selected. The same dynamic material parameters as identified for the fiber pullout experiments are assumed: for the matrix and the crack, $\rho_{0}=2100$ kg/m${}^{3}$ and, for the fiber, $\rho_{0}=980$ kg/m${}^{3}$, $\alpha^{I}=0.08$ and $\alpha^{\mathrm{II}}=0.51$. However, the values of $\alpha^{I}$ and $\alpha^{\mathrm{II}}$ should only be viewed as a rough approximation, as the experiments do not represent the same conditions as in the fully embedded fibers. In addition, the applied strain rate during the split Hopkinson bar experiment is significantly higher than during the single-fiber tension and fiber pullout tests. Therefore, the simulation does not lend itself to a quantitative comparison with the experimental data, but enables a qualitative parameter study.

**Figure 11:**
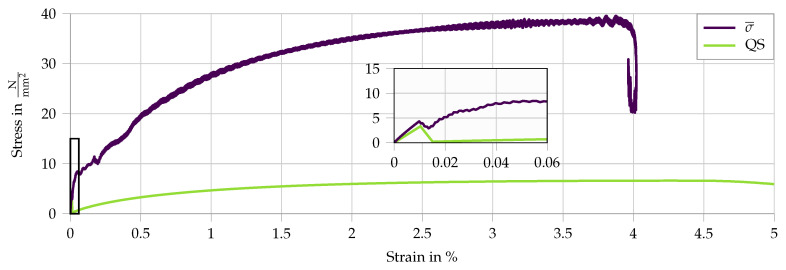
Results of the split Hopkinson tension bar simulation. The average of the stress–strain signals at both interfaces ($\bar{\sigma }$) compared to the quasi-static computation (QS). The zoom shows the initial cracking of the matrix.

The first results of the split Hopkinson bar simulation are presented in Figure 11. It compares the dynamic stress–strain curve to the quasi static results. The increase in stress level due to the dynamic conditions is evident. In addition, a shift is observed from the instantaneous fracture of all RVEs in the quasi-static case to a successive multiple cracking behavior, as also observed in the experiments. A comparison of failure patterns in the quasi-static and dynamic computation, as conducted in experimental tests, is not possible as such. With the applied homogenization technique the cracks only appear at the microscale. Due to the embedded fibers, they act structurally not as macroscopic cracks. The drop in stress at the end of the curve does not represent structural failure, which is further discussed in Section 5.3.3. To further understand the results and showcase the utility of the numerical framework, four parameter studies are conducted. Firstly, the two loading parameters are varied to understand the general influence of the loading conditions. Secondly, the influence of the strain rate sensitivity of the effective fiber pullout model is studied. Finally, the effect of the microscale inertia on the macroscopic measurements is presented. When comparing the dynamic numerical results in Figure 11 to the experimental results in Figure 7, it is evident that the dynamic increase does not agree accurately, as the simulated stress level is considerably higher. In addition to that, the experimental results show an earlier structural failure, which was not achieved in the simulations. This is due to the simplified fiber model in combination with the approximated microstructure.

#### 5.3.1. Parameter Study—$t_{\mathrm{cv}}$

**Figure 12:**
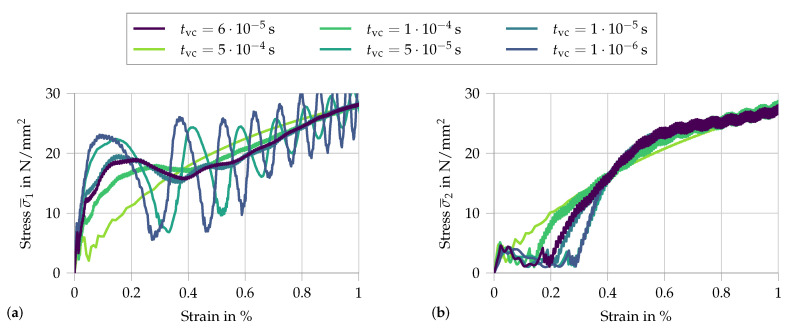
Analysis of the variation in rise time $t_{\mathrm{cv}}$ from $5\times 10^{-4}$ s to $10^{-6}$ s, with $v_{c}=3540$ mm/s: (**a**) the signal ${\bar{\sigma }}_{1}$ at the input face; and (**b**) the respective signal ${\bar{\sigma }}_{2}$ at the output face.

To study the influence of the applied loading function, the parameter $t_{\mathrm{vc}}$ is varied. A smaller value $t_{\mathrm{vc}}$ represents a faster rise time. This entails a higher acceleration. Therefore, this parameter allows visualizing the influence of the initial acceleration on the measured signal. To better understand the effects, the two signals ${\bar{\sigma }}_{1}$ and ${\bar{\sigma }}_{2}$ are each analyzed in a separate plot (see Figure 12).

With increasing acceleration, i.e., a shorter rise time $t_{\mathrm{vc}}$, the initial peak at the input face increases, as well as the subsequent macroscopic stress fluctuations. The only noticeable difference at the output face is a slight delay in stress increase for faster applied loads. This apparent delay is a simple result of the analyzed properties, as for a constant wave speed through the specimen the wave front will reach the output face at larger overall strains when the load is applied faster.

#### 5.3.2. Parameter Study—$v_{\mathrm{cv}}$

The other loading parameter is $v_{c}$. It controls the constant velocity during the impact wave. Increasing the velocity leads to a higher stress level during the loading pulse.

**Figure 13:**
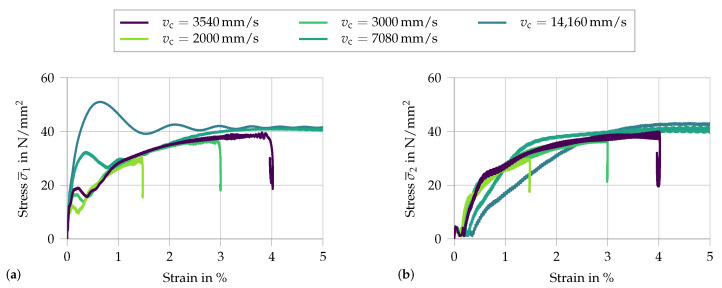
Analysis of the variation of $v_{c}$ from 2000 to 14,160 mm/s, with $t_{\mathrm{cv}}$ = $6\times 10^{-5}$ s: (**a**) the signal ${\bar{\sigma }}_{1}$ at the input face; and (**b**) the respective signal ${\bar{\sigma }}_{2}$ at the output face.

In addition to an increase in strain rate, the maximum acceleration during the initial phase increases, as the elevated speed is reached in the same time frame. The resulting stress–strain curves are depicted in Figure 13. The two effects discussed in the previous parameter study on $t_{\mathrm{cv}}$ are again observed. However, varying $v_{c}$ changes not only the initial loading phase but also the overall stress–strain curve at the loading face. Therefore, the first stress peak observed in Figure 13a is a combined result of the strain-rate sensitivity of the fibers and the macroscopic inertia. In addition, with increasing $v_{c}$, the stress equilibrium is reached only at higher strains. Another effect concerns the drop in stress at the end of the curves. This is not a global failure but rather the result of a decrease in strain rate, as is visible in the subsequent analysis. This parameter study demonstrates that, with a reduction in the rise time of the loading wave, the condition of dynamic stress equilibrium in the sample is violated and an experimental derivation of the material response based on the one-dimensional wave theory is not accurate.

#### 5.3.3. Parameter Study—Strain-Rate Sensitivity of the Fiber

**Figure 14:**
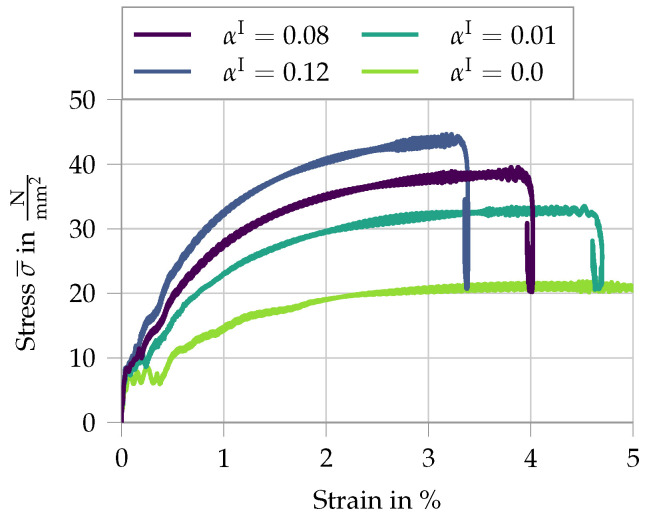
Analysis of the variation of the parameter $\alpha^{I}$ from 0 to 0.12.

After investigating the influence of the loading conditions on the obtained stress–strain curves, the dynamic influence of the microscale simulation on the macroscopic response is analyzed. First, the strain-rate sensitivity of the fiber is regarded. The results of the variation of $\alpha^{I}$ are given in Figure 14.

Here, the stress average $\bar{\sigma }$ of the two recorded stress signals is shown. As expected, with increasing strain-rate sensitivity of the fibers, the macroscopic stress level increases as well. From this analysis, it is evident that the previously observed drop in stress is due to a reduction in strain rate. This effect is due to a phase of a quasi-rigid translation of the specimen within the system, where the overall deformation does not change, but at the same time the strain rate decreases. For higher values of $\alpha^{I}$, the fibers are effectively stiffer, resulting in a higher wave speed though the specimen. Therefore, the phase of quasi-rigid translation is reached earlier.

#### 5.3.4. Parameter Study—Microinertia

**Figure 15:**
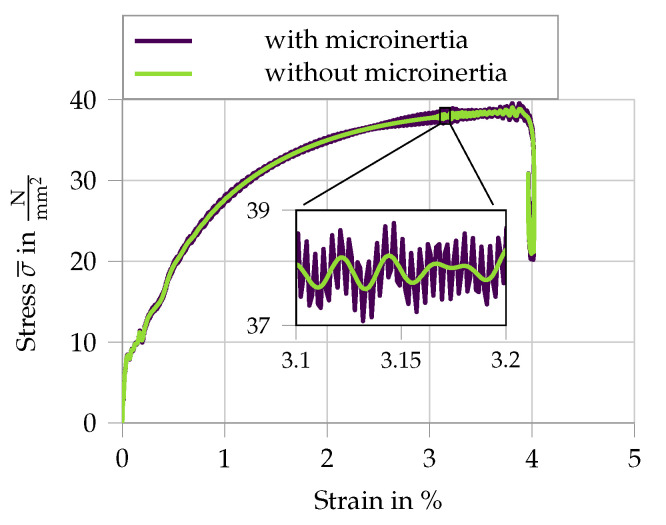
Analysis of the influence of microinertia on the macroscopic response.

Finally, the simulation is run without considering the inertia at the microscale compared to the full dynamic simulation, as presented in Figure 11. The average of the measured stress at the specimen interfaces is given in Figure 15, including a zoomed section to highlight the difference. For the presented microstructure, the overall macroscopic behavior does not appear to be significantly influenced by microscale inertia effects. This is not surprising, as the chosen RVE combined with the microscopic material models only allows for moderate dynamic activity. High frequency stress oscillations arising at the microscale once the crack has been formed are the results of the microcracks being able to freely open and close, as the fiber is anchored at the RVE boundary.

## 6. Conclusions and Outlook

This paper presents a numerical study involving a multiscale simulation of a split Hopkinson tension test on an SHCC specimen. The experimental loading conditions were replicated by approximating the loading pulse with a piece-wise polynomial function. To analyze the dynamic influence of various microscopic sources on the macroscale, such as the loading function, the strain-rate sensitivity of the embedded fiber, or inertia, a parameter study was conducted. The first observation is a substantial increase in specimen strength under impact. Compared to the experimental data, the stress increase as well as the ultimate strain exceeded the expected values. However, the simulation was able to replicate the phenomenon of multiple cracking, which could not be observed under quasi-static conditions due to the combination of a homogeneous stress state and the lack of variation in material properties. As expected, the variation of the loading wave, both for the increased rise time and the changed plateau values, show a significant influence on the measured signal. This showcases the need for numerical simulations accompanying dynamic experimental tests, to properly quantify the influence of inertia and achieve a better understanding of the pure material behavior. By changing the strain-rate sensitivity of the fiber, the extent of the influence on the overall stress was clearly visible. In addition to that, it allows inferring that the observed drop in stress is not a simulated failure of the specimen, but rather a phase of rigid translation, which results in a decreased strain rate. This effect has so far not been observed in the experiments as structural failure occurs before. The analysis of the influence of the microscale inertia showed for the chosen microstructure a negligible influence on the overall composite behavior. This does not necessarily indicate that microscopic inertia is irrelevant for SHCC under dynamic loading. It rather implies that more advanced micromechanical models need to be implemented before a final statement regarding the influence of microscopic inertia can be made. It is expected that a proper representation of crack propagation, combined with an improved fiber pullout simulation, will show a changing behavior under high strain rates. This will indirectly influence the macroscopic stress of the composite. Furthermore, there is the possibility that, for microstructures allowing for multiple cracks, the direct effect of microinertia will increase. More research is needed to give a definite answer. Nevertheless, the main insights from this numerical example are that this type of analysis is only possible by using a two-scale framework that includes the full inertia effects at the microscale. In conclusion, the utility of the multiscale homogenization framework for analyzing dynamic conditions is shown. However, improvements of the micromechanical models and further dynamic micromechanical experiments with fully embedded fibers are required to improve the prediction capacity of the SHCC simulation.

For future investigations, the micromechanical testing configurations could be adapted for higher displacement rates, such as presented in [35]. In addition, further micromechanical tests with fully embedded fibers could be conducted. This would allow for a more realistic assessment of the dynamic fiber tensile strength and fiber–matrix bond strength. More experimental data will enable an improved calibration of the developed material models. Moreover, the constitutive morphology, i.e., fiber distribution and orientation and flaw size distribution of SHCC, could be modeled based on statistical measures obtained by microtomography scans [36]. This would allow a more realistic simulation of the micromechanical and statistical influences on the multiple cracking process.
